# Outcomes of human umbilical cord blood-derived mesenchymal stem cells in enhancing tendon-graft healing in anterior cruciate ligament reconstruction: an exploratory study

**DOI:** 10.1186/s43019-021-00104-4

**Published:** 2021-09-16

**Authors:** Sang Won Moon, Sinhyung Park, Minkyung Oh, Joon Ho Wang

**Affiliations:** 1 Department of Orthopaedic Surgery, Inje University Haeundae Paik Hospital, Busan, Korea; 2 Department of Orthopaedic Surgery, Soonchunhyang University Hospital Bucheon, Gyeonggi-do, Korea; 3 Clinical Trial Center, Busan Paik Hospital, Inje University College of Medicine, Busan, Korea; 4 Department of Orthopaedic Surgery, Samsung Medical Center, Sungkyunkwan University School of Medicine, Seoul, 06351 Korea; 5 Department of Health Sciences and Technology and Department of Medical Device Management and Research, SAIHST, Sungkyunkwan University, Seoul, 06351 Korea

**Keywords:** Anterior cruciate ligament, Human umbilical cord blood-derived mesenchymal stem cell, Stem cell, Tendon graft healing, Healing, Randomized control trial

## Abstract

**Background:**

The study investigated whether allogeneic human umbilical cord blood-derived MSCs (hUCB-MSCs) could be safely used without treatment-related adverse events, reducing tunnel enlargement, and improve clinical results in human anterior cruciate ligament (ACL) reconstruction.

**Methods:**

Thirty patients were enrolled consecutively. They were divided into three groups by randomization. In the negative control group, ACL reconstruction surgery without additional treatment was performed. In the experimental group, a hUCB-MSC and hyaluronic acid mixture was applied to the tendon-bone interface of the femoral tunnels during ACL reconstruction surgery. In the positive control group, only hyaluronic acid was applied. Finally, 27 patients were analyzed after the exclusion of three patients. The incidence of treatment-related adverse events, clinical outcomes, including second-look arthroscopic findings, and the amount of tunnel enlargement, were evaluated.

**Results:**

There were no treatment-related adverse events in the treatment groups. Tunnel enlargement in the experimental group (579.74 ± 389.85 mm^3^) was not significantly different from those in the negative (641.97 ± 455.84 mm^3^) and positive control (421.96 ± 274.83 mm^3^) groups (*p* = 0.6468). There were no significant differences between the groups in clinical outcomes such as KT-2000 measurement (*p* = 0.793), pivot shift test (*p* = 0.9245), International Knee Documentation Committee subjective score (*p* = 0.9195), Tegner activity level (*p* = 0.9927), and second-look arthroscopic findings (synovial coverage of the graft, *p* = 0.7984; condition of the graft, *p* = 0.8402).

**Conclusions:**

Allogeneic hUCB-MSCs were used safely for ACL reconstruction without treatment-related adverse event in a 2-year follow-up. However, our study did not suggest any evidence to show clinical advantage such as the prevention of tunnel enlargement postoperatively and a decrease in knee laxity or improvement of clinical outcomes.

**Trial registration:**

CRIS, Registration Number: KCT0000917. Registered on 12 November 2013; https://cris.nih.go.kr/cris/index.jsp

## Background

Although anterior cruciate ligament (ACL) reconstruction has good clinical results, there are still several issues regarding the biological healing of ACL grafting as a cause of failed ACL reconstruction. Among these issues, the graft-tunnel interface is considered the most important target for the promotion of successful graft healing. Various biological agents have been evaluated in numerous studies to improve tendon-bone healing in the graft-tunnel interphase [1–14]. Autologous mesenchymal stem cells (MSCs) are the representative biological agents here. They can multiply, regenerate, and differentiate into various tissues. Animal studies have shown satisfactory results in autologous MSCs for ACL reconstruction [3, 5, 13, 15]. However, it requires two procedures (harvesting and application) for use, and the harvesting procedure, including bone marrow aspiration, is invasive. For that reason, allogeneic human umbilical cord blood-derived MSCs (hUCB-MSCs) are being used for cartilage regeneration due to the simplicity of a single procedure [16]. It is known that UCB-MSCs have high differential potential and tropism [17–19]. Additionally, hUCB-MSCs can be expanded and preserved [19]. Therefore, a one-stage procedure is possible.

In a previous animal study, the efficacy and safety of hUCB-MSCs in ACL reconstruction of a rabbit model have been reported [20]. Non-autologous transplantation of hUCB-MSCs was performed. There was no early immune rejection. Tendon-bone healing in the graft-tunnel interphase was improved through broad fibrocartilage formation. Femoral and tibial tunnel widening were decreased too.

In this study, based on the results from an animal model, we applied hUCB-MSCs to human ACL reconstruction. It was not allowed to do a biopsy for the histological evaluation. Therefore, tendon-bone healing was evaluated by measuring the extent of tunnel enlargement. The purpose of this study was to evaluated the safety of the allogeneic transplantation of hUCB-MSCs and contribution to improve tendon-bone healing in the graft-tunnel interphase during ACL reconstruction. We hypothesized that allogeneic hUCB-MSCs could be safely used, decrease tunnel widening, and enhance the clinical results in ACL reconstruction.

## Methods

### Study designs and participants

Between January 2014 and April 2014, eligible patients, who were between 20 and 50 years old with isolated ACL rupture, were included in this study. Anterior cruciate ligament rupture was diagnosed by physical examination (Lachman test grade II or III and pivot shift test grade I, II, or III) and magnetic resonance imaging. Participants were excluded based on the following criteria: diagnosis of degenerative osteoarthritis of the knee, revision or history of other surgeries including stem cell treatment, any infectious disease, autoimmune disease, or serious medical disease, pregnancy and breastfeeding, psychiatric disorder, epilepsy, and alcohol abuse or smoking.

**Fig. 1:**
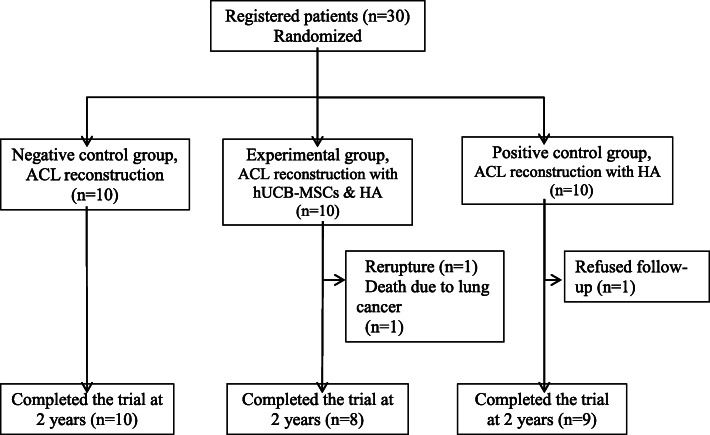
Consolidated Standards of Reporting Trials (CONSORT) flow diagram

Thirty patients were enrolled in this study consecutively and prospectively randomized into three treatment groups: negative control (*n* = 10), experimental (*n* = 10), and positive control (*n* = 10) groups. In the negative control group, ACL reconstruction surgery without any additional treatment was performed. In the experimental group, a hUCB-MSC and hyaluronic acid mixture (hUCB-MSCs & HA) was applied on the tendon-bone interface of the femoral and tibial tunnels during ACL reconstruction surgery. In the positive control group, only HA was applied. Three patients did not complete the follow-up evaluations. In one patient in the experimental group, the ACL re-ruptured due to minor trauma at 6 months after surgery, and another patient in the experimental group died from lung cancer at 23 months after surgery. Moreover, one patient in the positive control group refused follow-up. Therefore, 27 patients were included in the final analysis (Fig. 1).

### Randomization and blinding

A biostatistician created a randomization sequence using the SAS program for a random permuted block design with block size of 3. Each treatment group was randomly allocated in a 1:1:1 ratio in each block. The investigator assigned the subjects to one of the three treatment groups in the order in which they were enrolled. This study was conducted single blind so that the subjects could not know to which treatment group they were assigned.

### Preparation of hUCB-MSCs

The hUCB-MSCs & HA (Cartistem®; Medipost, Seongnam-si, Gyeonggi-do, Korea, http://www.medi-post.com) were produced by Medipost. This was approved by the regulatory authority. Human umbilical cord blood was collected and hUCB-MSCs were isolated and characterized according to previously published methods [21]. The hUCB-MSCs & HA product was composed of a vial of 7.5 × 10^6^ hUCB-MSCs and a vial of 60 mg HA hydrogel. The composite was made in the operating room by mixing the hUCB-MSCs and the HA.

### Surgical protocol

Portal formation was conducted in the usual manner. Arthroscopic examinations were performed. The hamstring tendon was harvested, and double-bundle ACL reconstruction was performed using the method described in the previous study [22–24].

### Application of the stem cell-based medicinal product

**Fig. 2:**
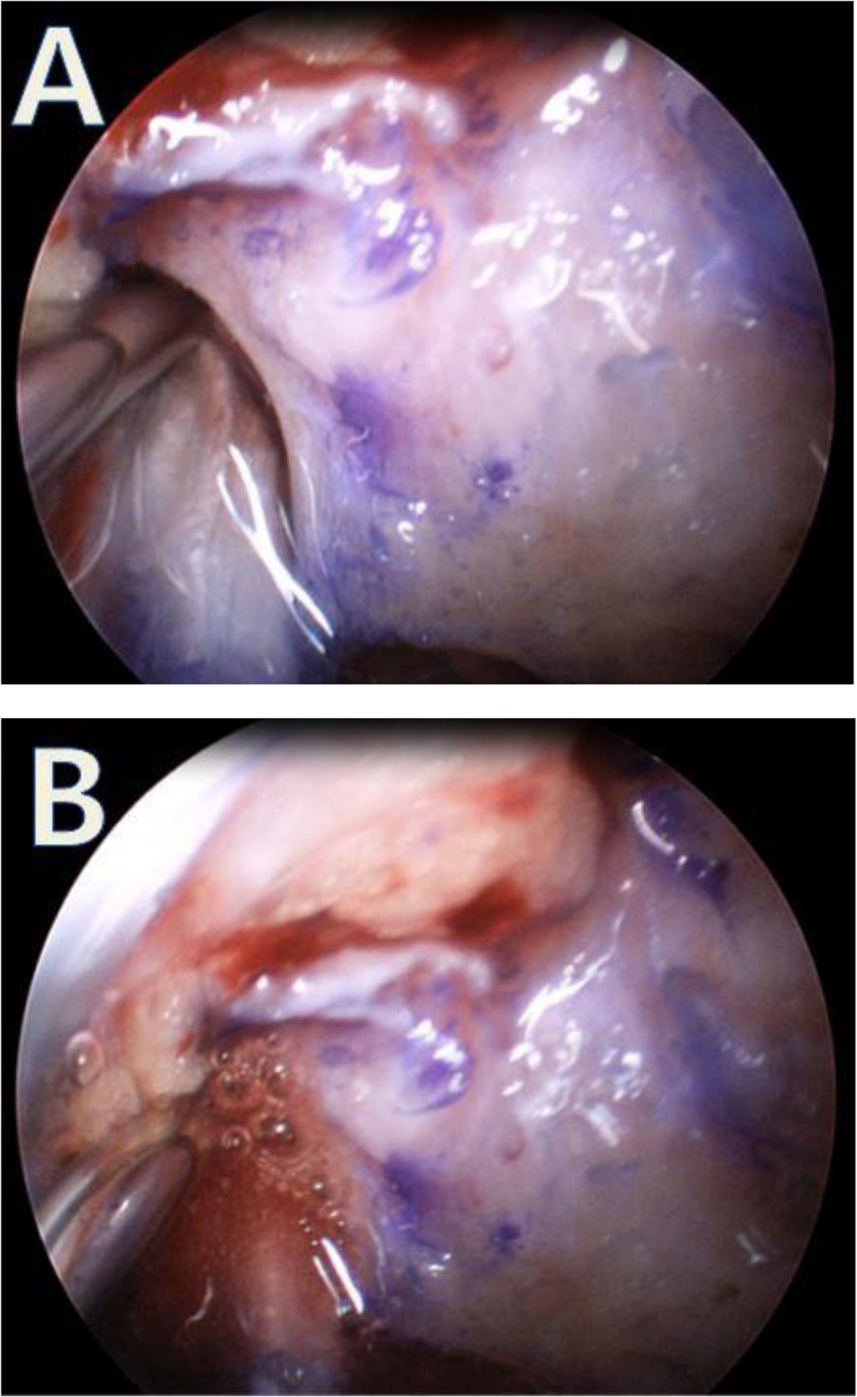
A composite of allogeneic human umbilical cord blood-derived mesenchymal stem cells (hUCB-MSCs) and hyaluronic acid hydrogel was injected into the femoral tunnels under arthroscopic guidance. **A** The needle tip was inserted into the femoral tunnel under arthroscopic guidance. **B** A positioned composite of allogeneic hUCB-MSCs and hyaluronic acid hydrogel was injected into the femoral tunnels after draining the fluid

Anterior cruciate ligament reconstruction was performed, and intra-articularly administered saline was removed to dry the circumference of the knee joint. In the experimental group, hUCB-MSCs & HA were injected on to the tendon-bone interface of the femoral tunnels under arthroscopic guidance (Fig. 2). In the positive control group, only HA was injected.

### Rehabilitation protocol

Patients in the three groups underwent the same rehabilitation protocol. All patients performed active quadriceps isometric exercises and range of motion exercises immediately after surgery. Gradual motion was permitted with a limited motion brace. Range of motion was increased by 15° per week. At 6 weeks after surgery, full ranges of motion were permitted. At 6 months after surgery, straight-line running was permitted.

### Computed tomography (CT) protocol and measurements

**Fig. 3:**
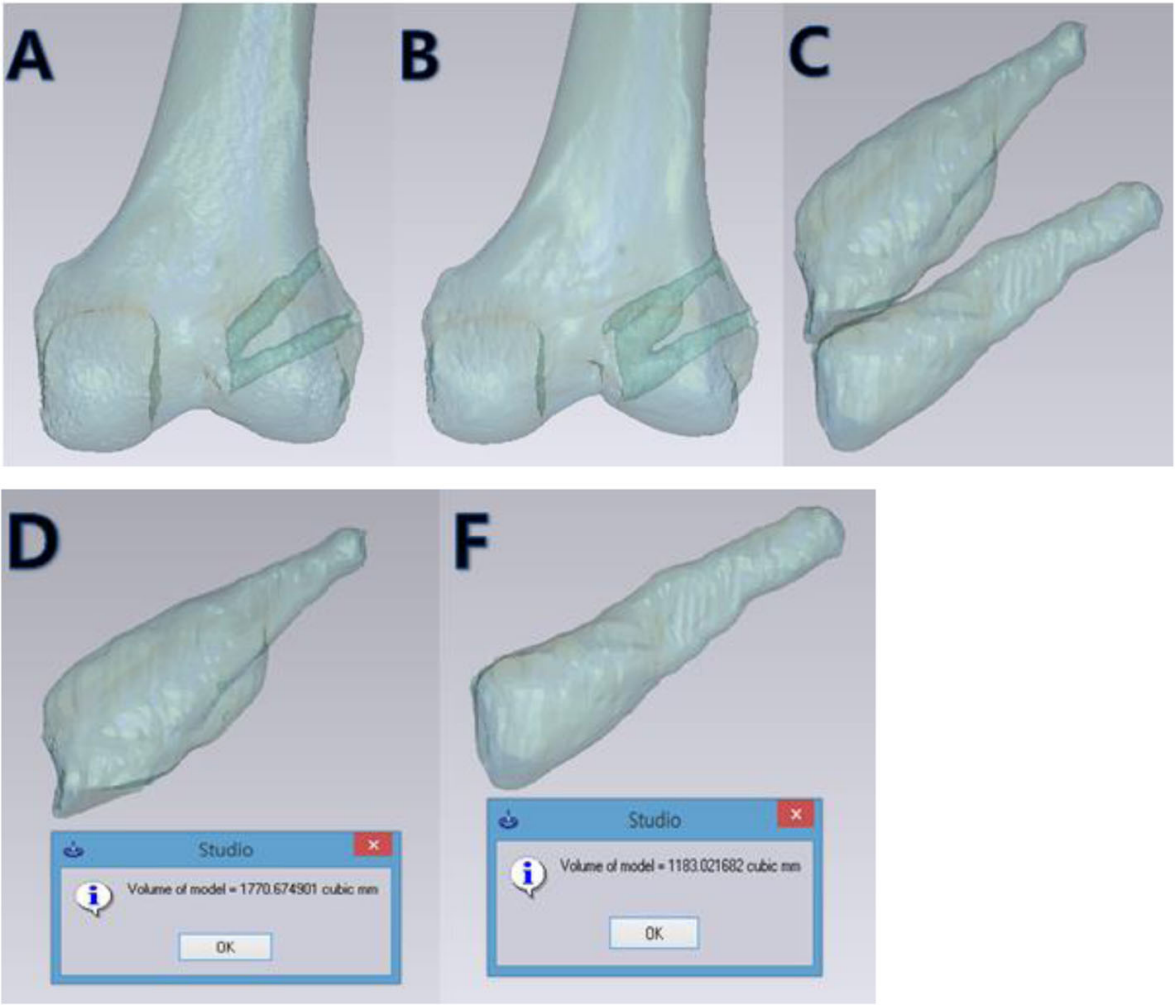
The volumes of three-dimensional (3D)-reconstructed models were measured using Mimics software. **A** Transparent image obtained through a 3D-reconstructed model of the femur showed the AM and PL tunnel postoperatively. **B** The widening of the tunnel was clearly observed at 12 months, especially in the AM tunnel. **C** The tunnel was separated from the 3D-reconstructed model. **D**, **F** The volume of the AM and PL tunnel was estimated using Mimics software

Computed tomography (CT) was performed 1 day and 12 months after ACL reconstruction. The CT scanner LightSpeed VCT (GE Medical Systems, Milwaukee, WI, USA) was used in all examinations. Computed tomography was performed in the full extended knee position. The collimation was 16 × 0.625 mm. The tube parameters were 120 kVp and 200 mA. The acquisition matrix was 512 × 512. The field of view was 140 mm, and the slice thickness was 0.625 mm. The Digital Imaging and Communications in Medicine data were extracted by a picture archiving and communication system and exported to Mimics software (Materialise, Leuven, Belgium). Three-dimensional (3D) reconstruction of the femur was generated using the “Thresholding,” “Region Growing,” “Edit Mask,” and “Calculate 3D” functions. To separate the femoral tunnel from other areas, “Multiple Slice Edit” and “Boolean Operations” functions were used. Three-dimensional reconstructed models of the tunnel were generated by the “Calculate 3D” function. The volumes of the 3D-reconstructed models were measured by Mimics software (Fig. 3).

### Outcomes

The primary outcomes were safety and tunnel enlargement. Safety parameters included vital signs, laboratory tests (hematology, blood chemistry, blood coagulation, electrolyte levels, inflammation parameters, and a urine test), and treatment-related adverse events. Treatment-related adverse events in the knee were assessed by assessing swelling, tenderness, active range of motion, and pain. Other adverse events were categorized according to the World Health Organization Common Toxicity Criteria for Adverse Events [25]. The tunnel enlargement was evaluated by tunnel volume change at 12 months after surgery.

The secondary outcomes were clinical outcomes. These were evaluated with a KT-2000 arthrometer (MEDmetric Corp, San Diego, CA, USA) side-to-side laxity measurement (KT-2000), the Lachman test, pivot shift test, International Knee Documentation Committee (IKDC) subjective knee evaluation form score, Lysholm score, Knee injury and Osteoarthritis Outcome Score (KOOS), and the Tegner activity level. The safety and clinical outcomes were assessed preoperatively and at 6, 12, and 24 months. An independent research assistant administered the questionnaires on safety and efficacy.

### Second-look arthroscopy

Second-look arthroscopy was recommended in all patients as planned in the clinical trial at 12 months after surgery. The risks and benefits of second-look arthroscopy were explained. Any tumor formation was evaluated around the site where the hUCB-MSCs were injected. Synovial coverage of the grafts was divided into good (synovial coverage of > 80% of the graft), fair (50 to 80% coverage), or poor (< 50% coverage) [26, 27]. The condition of each graft was classified as normal or damaged. Any graft showing an apparent lack of tension or a significant tear of one or more tendon strands was considered damaged.

### Statistical analysis

Data were presented as mean ± standard deviation for continuous variables and as count and percentage for categorical variables. An analysis of variance (ANOVA) or Kruskal-Wallis test was conducted to compare the means of the three groups. A repeated measures ANOVA was used in analyzing the effects of time and treatment and their interaction. A *p* value < 0.05 was considered statistically significant. All statistical analyses were performed using SAS 9.4 (SAS Institute, Cary, NC, USA).

## Results

### Baseline characteristics

Preoperative demographic data are presented in Table 1. The mean age of the 27 subjects was 35.7 years (range, 20 to 50 years). No significant differences in baseline demographics were found among the groups.

**Table 1:** Demographics of the participants by group

	Negative control group (*n* = 10)	Experimental group (*n* = 8)	Positive control group (*n* = 9)	*p* value
Age, years, mean ± SD (range)	38 ± 5.72 (26–44)	33.25 ± 10.95 (20–50)	37.11 ± 6.7 (29–49)	0.4284
Sex, male/female, *n* (percentage)	5 (18.52)/5 (18.52)	6 (22.22)/2 (7.41)	6 (22.22)/3 (11.11)	0.6111
Height (cm), mean ± SD (range)	167.54 ± 8.21 (153.5–180)	172.45 ± 9.66 (155.1–183.1)	173.1 ± 9.57 (156.2–186)	0.3627
Weight (kg) mean ± SD (range)	70.63 ± 11.94 (56–91.5)	77.77 ± 20.04 (51.4–110.9)	74.13 ± 11.99 (54.5–91.2)	0.6003
Body mass index (kg/m^2^), mean ± SD (range)	25.13 ± 3.58 (18.52–31.55)	25.81 ± 4.59 (21.3–33.08)	24.62 ± 2.45 (20.97–27.58)	0.7927

*SD* standard deviation

### Safety

Vital signs, laboratory tests, and physical examination (swelling and tenderness) were within the normal range in all treatment groups. There were no significant differences in active range of motion (*p* = 0.92) and visual analog pain scale (*p* = 0.8694) among the groups. No infection was observed after surgery in all the groups. Tumor formation was not found on second-look arthroscopy. No patient withdrew from the study due to treatment-related adverse events. However, one patient in the experimental group died due to lung cancer. He was diagnosed with multiple bullae in both lung apices about 1 year before enrollment in this study. He was treated just with observation and annual follow-up by a pulmonologist. There was no interval change in follow-up evaluation. About 5 months after second-look arthroscopy, he visited other hospitals due to sputum production and was diagnosed with lung cancer. However, we could not find any relevance to hUCB-MSCs.

### Tunnel enlargement

Table 2 shows the result of tunnel volume measurements. Mean differences in femoral tunnel volume (AM + PL) between postoperation and 12 months were not significantly different among the treatment groups although the difference was widest in the control group compared to the other treatment groups.

**Table 2:** Tunnel volume measurements (mm^3^)

		Postoperative	12 months	Difference	*p* value
Femoral tunnel (AM + PL), mean ± SD	Negative control group (*n* = 10)	1052.87 ± 265.84	1694.84 ± 540.36	641.97 ± 455.84	0.6468
	Experimental group (*n* = 8)	1001.19 ± 258.27	1580.93 ± 525.78	579.74 ± 389.85	
	Positive control group (*n* = 9)	919.08 ± 278.34	1341.04 ± 491.61	421.96 ± 274.83	
AM tunnel mean ± SD	Negative control group (*n* = 10)	645.23 ± 157.67	1042.93 ± 369.88	397.7 ± 272.18	0.2601
	Experimental group (*n* = 8)	701.36 ± 220.58	968.82 ± 333.31	267.46 ± 250.56	
	Positive control group (*n* = 9)	584.84 ± 130.25	791.76 ± 310.76	206.92 ± 229.37	
PL tunnel mean ± SD	Negative control group (*n* = 10)	407.64 ± 126.64	651.91 ± 321.11	244.27 ± 315.52	0.3677
	Experimental group (*n* = 8)	299.83 ± 104.65	612.11 ± 292.12	312.28 ± 218.23	
	Positive control group (*n* = 9)	334.24 ± 174.89	549.23 ± 288.04	215.04 ± 145.52	

*AM* anteromedial, *PL* posterolateral, *SD* standard deviation

### Clinical outcomes

All clinical parameters (KT-2000, Lachman test, pivot shift test, IKDC subjective score, Lysholm score, KOOS, and Tegner activity level) were significantly improved through the 2-year follow-up compared to preoperative measurements (*p* < 0.001) (Table 3). Although anterior stability measured by the KT-2000 arthrometer, the IKDC subjective score, and Tegner activity level tended to improve more in the experimental group than in the negative control group, all clinical parameters showed no significant differences between the 2-year follow-up and the treatment groups. In the only treatment groups, the KT-2000 measurement was significantly different (*p* = 0.010).

**Table 3:** Clinical outcome measurements

		Preoperative	6 months	12 months	24 months	Time/group	Time × group
KT-2000 arthrometer measurement, mean ± SD	Negative control group (*n* = 10)	3.4 ± 2.13	0.78 ± 1.84	1 ± 1.03	1.92 ± 0.92	< .0001/0.0109	0.793
	Experimental group (*n* = 8)	5.1 ± 1.45	2.38 ± 1.66	1.5 ± 1.78	1.8 ± 2.05		
	Positive control group (*n* = 9)	4.3 ± 2.18	2.25 ± 1.6	2.39 ± 1.39	2.13 ± 1.13		
Lachman test^a^	Negative control group (*n* = 10)	2.4 ± 0.7	0 ± 0	0.22 ± 0.44	0.29 ± 0.76	< .0001/0.8681	0.8038
	Experimental group (*n* = 8)	1.95 ± 0.9	0.14 ± 0.38	0.5 ± 0.93	0.4 ± 0.89		
	Positive control group (*n* = 9)	2.2 ± 0.79	0.1 ± 0.32	0.38 ± 1.06	0.56 ± 1.01		
Pivot shift test^a^	Negative control group (*n* = 10)	1.3 ± 0.48	0 ± 0	0.13 ± 0.35	0.2 ± 0.45	< .0001/0.2199	0.9245
	Experimental group (*n* = 8)	1.4 ± 0.97	0.2 ± 0.45	0.14 ± 0.38	0.4 ± 0.89		
	Positive control group (*n* = 9)	1.3 ± 0.48	0.4 ± 0.84	0.63 ± 1.19	0.67 ± 1.12		
International Knee Documentation Committee subjective score	Negative control group (*n* = 10)	56.2 ± 17.7	68.19 ± 15.14	79.95 ± 8.37	76.88 ± 21.52	< .0001/0.2813	0.9195
	Experimental group (*n* = 8)	50.88 ± 14.86	70.11 ± 16.94	77.25 ± 18.75	87.36 ± 14.93		
	Positive control group (*n* = 9)	45.04 ± 19.13	65.96 ± 21.7	75.04 ± 16.69	74.2 ± 22.67		
Lysholm score	Negative control group (*n* = 10)	66.6 ± 19.63	89.22 ± 8.96	91.33 ± 5.02	88.44 ± 13.87	< .0001/0.1384	0.8227
	Experimental group (*n* = 8)	73.8 ± 12.87	82.5 ± 23.11	92.63 ± 8.09	95 ± 6.38		
	Positive control group (*n* = 9)	62.7 ± 20.92	87 ± 9.27	87.89 ± 11.04	83.9 ± 25.71		
Knee Injury and Osteoarthritis Outcome Score	Negative control group (*n* = 10)	66.55 ± 20.14	87.95 ± 7.06	93.78 ± 3.19	95.6 ± 4.99	< .0001/0.9053	0.8299
	Experimental group (*n* = 8)	74.17 ± 13.05	86.56 ± 13.77	92.45 ± 10.43	95.08 ± 9.41		
	Positive control group (*n* = 9)	76.14 ± 17.58	90.63 ± 9.08	90.5 ± 11.27	92.43 ± 12.97		
Tegner activity level	Negative control group (*n* = 10)	3.7 ± 2.16	4.89 ± 1.27	5.89 ± 1.76	5.89 ± 2.42	< .0001/0.6755	0.9927
	Experimental group (*n* = 8)	3.9 ± 1.45	4.88 ± 0.64	6.38 ± 2.5	6.57 ± 1.72		
	Positive control group (*n* = 9)	3.3 ± 1.34	4.9 ± 1.97	5.89 ± 1.62	5.8 ± 2.1		

^a^Lachman test/pivot shift test +, ++, +++ converted to 1, 2, 3

### Second-look arthroscopy

**Fig. 4:**
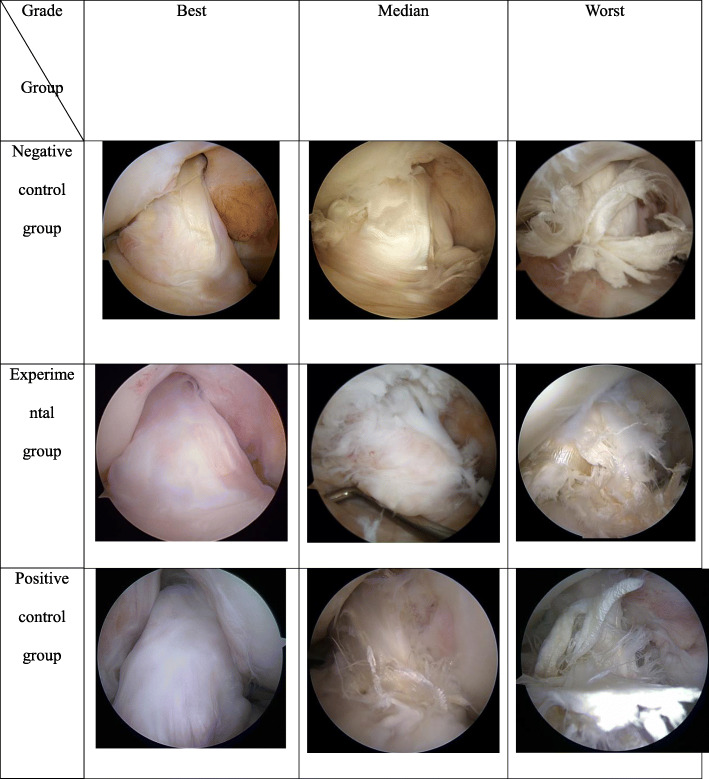
The second-look arthroscopic findings of each group were divided into best, median, and worst

Twenty-five patients agreed and two patients refused (one patient in the control group and one patient in the positive control group) to undergo second-look arthroscopy. Figure 4 shows the best, median, and worst second-look arthroscopic findings of each group. However, there was no significant difference in synovial coverage and condition of the graft among the three groups (Table 4).

**Table 4:** Second-look arthroscopic findings

	Negative control group	Experimental group	Positive control group	*p* value
Synovial coverage of the graft				0.7984
Good	4	4	5	
Fair	2	1	2	
Poor	3	3	1	
Condition of the graft				0.8402
Normal	5	4	5	
Damaged	4	4	3	

## Discussion

The principal finding of this study was that allogeneic hUCB-MSCs can be used safely without treatment-related adverse events in a 2-year follow-up. However, this study did not suggest evidence of any clinical advantages such as the prevention of tunnel enlargement postoperatively, decrease in knee laxity or improvement in other clinical outcomes.

Reconstruction of an ACL is a predictably successful surgical procedure to restore anterior stability. However, the graft failure rate after ACL reconstruction was 14% [28]. Many causes of graft failure after ACL reconstruction have been suggested, such as malpositioned tunnels, improper tensioning, and failure of graft fixation and maturation. To promote better graft maturation, numerous studies have focused on bone-tendon healing after ACL reconstruction. To improve bone-tendon healing, several biological agents were proposed in both in vitro and in vivo trials. Platelet-rich plasma and MSCs are representative biological agents. Especially, MSCs from different sources have recently been proposed to enhance bone-tendon healing after ACL reconstruction. The sources of MSCs are divided into two types, autologous and allogeneic. Autologous MSCs have been thought to be the optimal type but have the disadvantage of requiring an additional procedure. Therefore, allogeneic MSCs can be an attractive alternative without the need for another procedure.

Human umbilical cord blood-derived mesenchymal stem cells are a good source of allogeneic MSCs because of their availability, high proliferation capacity, and low immunogenicity [29]. The safety profile of hUCB-MSCs was proved in over 7 years of follow-up in a previous study [16]. Our previous study reported that hUCB-MSCs were applied safely in ACL reconstruction of a rabbit model and enhanced bone-tendon healing through fibrocartilage formation. Femoral and tibial tunnel widening decreased compared with that in the control group [20]. This study did not suggest any significant interaction between time and groups. Only the KT-2000 measurement was significantly different in the treatment groups (*p* = 0.010). This means that anterior stability significantly improved in the experimental group. However, other clinical measurements were not significantly different among groups. Therefore, this study is insufficient to suggest the efficacy of using hUCB-MSCs in ACL reconstruction.

In a previous study, filling the bone tunnel with HA improved bone-tendon healing in ACL reconstruction in a rabbit model [30]. In an animal model, the effect of HA was reported and, hence, a positive control group was included in this study. It is important to distinguish the efficacy of HA and hUCB-MSCs on bone-tendon healing. However, the positive control group had significantly more improvement in anterior stability than the negative control group.

The limitations of this study should be considered. First, a small number of patients participated in this clinical trial. A small sample size is an inherent limitation of this type of human clinical trial because the risk-benefit ratio is unknown. This study was the first human clinical trial that applied hUCB-MSCs in the bone tunnel during ACL reconstruction. Second, we did not evaluate any histology results. Additionally, it was unclear whether the fibrocartilage zone formed as expected in the experimental group. This is an important parameter in interpreting the results of the study. However, a biopsy for histological evaluation was not allowed. Tunnel enlargement was an indirect parameter to evaluate fibrocartilage zone formation. In a previous animal study, enhanced tendon-bone healing through broad fibrocartilage formation decreased tunnel widening [20]. Therefore, tendon-bone healing was evaluated through tunnel enlargement.

## Conclusions

Allogeneic hUCB-MSCs were applied safely for ACL reconstruction without treatment-related adverse events in a 2-year follow-up. However, this study did not suggest any evidence to show clinical advantages such as the prevention of tunnel enlargement postoperatively and decrease in knee laxity or improvement in clinical outcomes.

### Potential conflict of interest

The authors declare that that they have no conflicting financial interests.

## Data Availability

Not applicable.

## Abbreviations

ACL: Anterior cruciate ligament
MSCs: Mesenchymal stem cells
hUCB-MSCs: Allogeneic human umbilical cord blood-derived MSCs
